# Impact of initial respiratory compliance in ventilated patients with acute respiratory distress syndrome related to COVID-19

**DOI:** 10.1186/s13054-020-03133-9

**Published:** 2020-07-09

**Authors:** Florent Laverdure, Amélie Delaporte, Astrid Bouteau, Thibaut Genty, François Decailliot, François Stéphan

**Affiliations:** 1 Department of Anesthesiology and Intensive Care, Hôpital Marie Lannelongue, Groupe Hospitalier Paris Saint Joseph, Paris, France; 2 Pediatric Intensive Care Unit, Hôpital Marie Lannelongue, Groupe Hospitalier Paris Saint Joseph, Paris, France

**Keywords:** COVID-19, Acute respiratory distress syndrome, Invasive mechanical ventilation, Respiratory compliance, Prone positioning, Positive end-expiratory pressure

Dear Editor,

Coronavirus disease 2019 (COVID-19) is associated with a high fatality rate in patients requiring invasive mechanical ventilation (IMV) [1]. COVID-19-related acute respiratory distress syndrome (COVID-ARDS) might exhibit vascular insults, resulting in loss of hypoxic pulmonary vasoconstriction, and subsequent dissociation between profound hypoxemia and preserved static compliance of the respiratory system (Cst-rs) [2]. Experts recently distinguished two phenotypes of COVID-ARDS according to their Cst-rs [2]: patients were classified as groups L (low elastance (or high Cst-rs) and low recruitability) and H (high elastance and high recruitability). They recommended different ventilatory approaches [3], contrary to Sepsis Surviving Campaign guidelines [4]. We describe characteristics and outcomes in patients with different initial Cst-rs, but all receiving IMV following ARDS guidelines.

We report the courses of respiratory parameters and outcomes in an observational cohort of 36 patients who developed COVID-ARDS requiring IMV from March 17 to April 18, 2020. Patients were divided into two groups (low and high Cst-rs) according to their initial Cst-rs was above or below the median value. We applied institutional ARDS procedures to all patients. Our management was based on the systematic use of neuromuscular blockers for at least 48 h, positive end-expiratory pressures (PEEP) titrated on oxygenation, and prone positioning sessions if PaO_2_/FiO_2_ ratio dropped below 150. Patients’ data were analyzed until patients were discharged from the intensive care unit or died. Courses of Cst-rs, PEEP, and tidal volumes were analyzed using a linear mixed model.

The median baseline Cst-rs was 36 mL/cmH_2_O [interquartile range (IQR) 29–44]. Characteristics of the patients at baseline, therapeutic interventions, and outcome measures are provided in Table 1. Twenty-nine patients (80.6%) in whom PaO_2_/FiO_2_ ratio dropped below 150 were placed in prone position. Courses of Cst-rs, PEEP levels, and tidal volumes are provided in Fig. 1. Cst-rs did not vary over time in both groups and remained higher in the high Cst-rs group (mean difference 17.7 mL/cmH_2_O [95% CI 11.3–24.0] compared to the low Cst-rs group, *P* < 0.001). Tidal volumes were higher in the high Cst-rs group (mean difference 0.90 mL/kg [95% CI 0.31–1.50] compared to the low Cst-rs group, *P* = 0.005). PEEP levels were not different between groups and decreased over time.

**Table 1:** Baseline characteristics, therapeutic interventions, and outcomes of patients, according to respiratory compliance

	Overall (***N*** = 36), no. (%) of patients^**a**^	High respiratory compliance (***N*** = 17), no. (%) of patients^**a**^	Low respiratory compliance (***N*** = 19), no. (%) of patients^**a**^	***P*** value between groups
**Baseline characteristics**				
Age, mean ± SD, years	53.4 ± 10.2	56.1 ± 7.1	50.9 ± 12.1	0.12
Male sex	30 (83.3)	16 (94.1)	14 (73.7)	0.18
Obesity^b^	14 (38.9)	3 (17.7)	11 (57.9)	**0.02**
Diabetes mellitus	11 (30.6)	5 (29.4)	6 (31.6)	1.0
Arterial hypertension	16 (44.4)	11 (64.7)	5 (26.3)	0.19
SAPS 2 score, median [IQR]	31 [27–36]	31 [29–36]	29 [22–39]	0.51
SOFA score, median [IQR]	5 [4–7]	6 [4–7]	4 [3–6]	**0.04**
Tidal volume, mean ± SD, mL/kg	6.1 ± 0.6	6.2 ± 0.3	6.0 ± 0.7	**0.02**
Respiratory frequency, median [IQR], breaths/min	25 [24–27]	25 [24–26]	26 [24–28]	0.64
FiO_2_, median [IQR], %	65 [50–100]	60 [40–80]	70 [60–100]	0.07
PaO_2_/FiO_2_ ratio, median [IQR]	152 [112–240]	209 [150–256]	117 [83–201]	**0.02**
PEEP, mean ± SD, cmH_2_O	13.4 ± 3.2	13.4 ± 3.6	13.4 ± 3.1	0.92
Respiratory compliance, mean ± SD, mL/cmH_2_O^c^	39.4 ± 16.9	51.8 ± 16.4	28.3 ± 6.1	
**Therapeutic interventions**				
Prone positioning	29 (80.6)	12 (70.6)	17 (89.5)	0.22
Number of sessions, median [IQR]	4.0 [2.0–6.0]	4.0 [2.5–5.0]	5.0 [1.7–6.0]	0.91
Inhaled nitric oxide	9 (25.0)	2 (11.8)	7 (36.8)	0.13
Venovenous ECMO	7 (19.4)	0 (0.0)	7 (36.8)	**0.008**
Vasopressors	31 (86.1)	17 (100.0)	14 (73.7)	0.048
Renal replacement therapy	7 (19.4)	3 (17.7)	4 (21.1)	1.0
Hydroxychloroquine	32 (88.9)	16 (94.1)	16 (84.2)	0.6
Steroïds	11 (30.6)	5 (29.4)	6 (31.6)	1.0
**Outcomes**				
Ventilator-free days, median [IQR]	3.0 [0.0–14.5]	10.0 [0.0–17.2]	0.0 [0.0–8.0]	**0.04**
Mortality at day 28	4 (11.1)	1 (5.9)	3 (15.8)	0.61

*Abbreviations*: *ECMO* extracorporeal membrane oxygenation, *FiO*_*2*_ fraction of inspired oxygen, *PEEP* positive end-expiratory pressure

^a^Unless otherwise indicated

^b^Obesity is defined by a body mass index above 30 kg/m^2^. The formula for body mass index is weight in kilograms divided by height in meters squared

^c^No statistical comparison performed

**Fig. 1:**
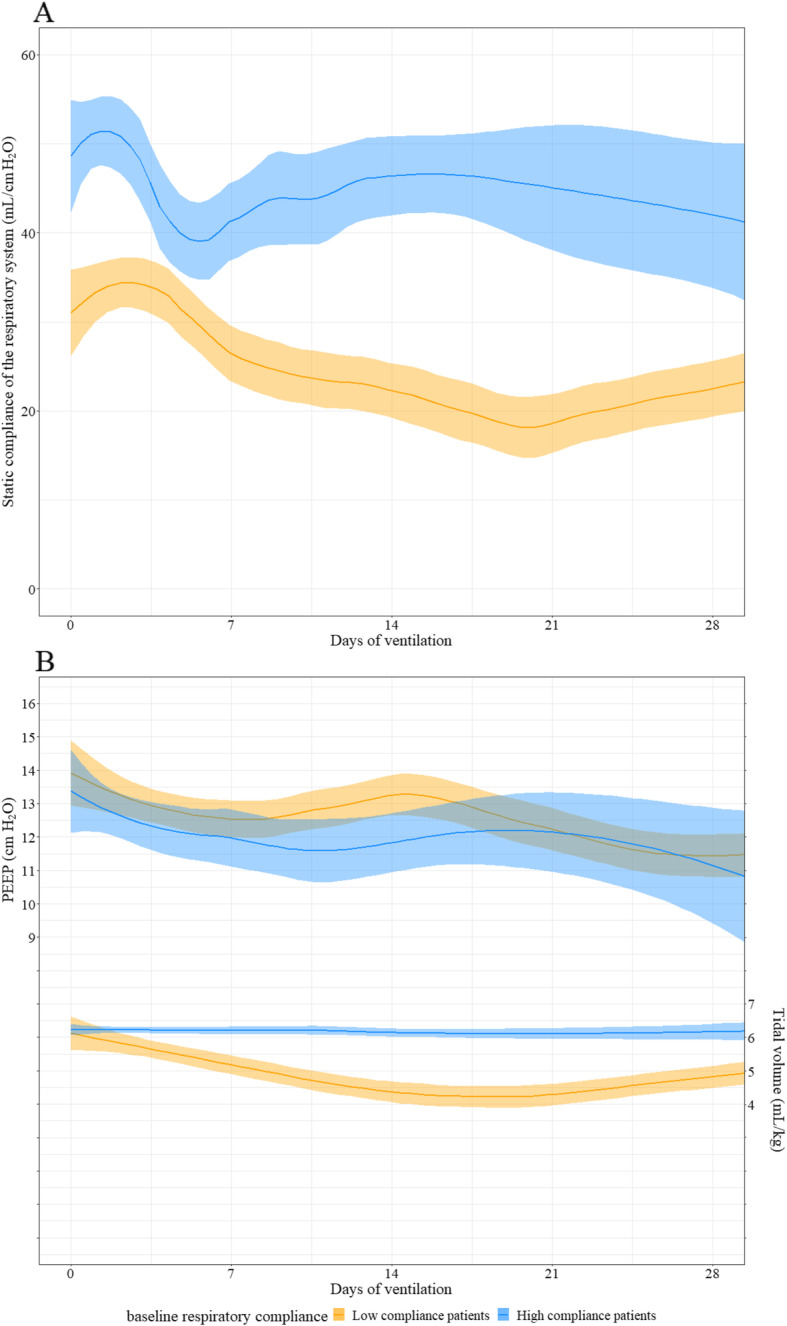
Course of the respiratory system static compliances (Cst-rs), positive end-expiratory pressures (PEEP), and tidal volumes (Vt). The means and 95% confidence intervals are represented respectively by solid lines and colored areas. Results are expressed in mean differences [95% CI]. **a** Cst-rs remained higher in the high initial Cst-rs group. There was no significant effect of time on Cst-rs (slope = − 0.03 mL/cmH_2_O/day of ventilation [95% CI − 0.17 to 0.12], *P* = 0.70). **b** PEEP levels did not differ between groups (high vs. low Cst-rs group − 0.69 cmH_2_O [95% CI − 2.05 to 0.66], *P* = 0.33). There was a statistically significant effect of time on PEEP (slope = − 0.10 cmH_2_O/day of ventilation [95% CI − 0.13 to − 0.06], *P* < 0.001). Vt were higher in the high Cst-rs group. There was no significant effect of time on Vt (slope = − 0.006 [− 0.02 to 0.007], *P* = 0.375)

On day 28, 32 patients (88.9%) survived and 25 (69.4%) were discharged from the intensive care unit. As of May 30, 2020, weaning from mechanical ventilation was effective in 16 high Cst-rs patients (94.1%) and 13 low Cst-rs patients (68.4%) (*P* = 0.09).

As previously suggested [3], some COVID-ARDS patients exhibit high initial Cst-rs. However, the median baseline Cst-rs was not different from Cst-rs observed in “typical” non-COVID-ARDS, as demonstrated in another study [5]. The high Cst-rs did not drop and remained different from the initial low Cst-rs during the first 28 days, suggesting a lack of transition from a high to a low Cst-rs phenotype in patients receiving neuromuscular blockers. We therefore hypothesize that if this transition exists, self-inflicted lung injury during spontaneous ventilation or asynchronies is one of its main determinants.

Although therapeutic management of low Cst-rs patients is not disputed [2, 6], a low-PEEP, high-FiO_2_, liberal tidal volume approach has been suggested for high Cst-rs patients. Using established ARDS therapies [3] with either low or high Cst-rs, the survival rate is better than initially reported [1], following a recent publication using the same strategy [5]. A low initial Cst-rs could be a marker of severity, as suggested by more extracorporeal membrane oxygenation requirement and less ventilator-free days at day 28.

Limitations include the small number of patients and the retrospective design. While further study is needed, our findings provide arguments to treat all COVID-ARDS with established ARDS therapies, whatever the initial value of Cst-rs.

## Data Availability

The datasets used and analyzed during the current study are available from the corresponding author on reasonable request.

## Abbreviations

ARDS: Acute respiratory distress syndrome
COVID-19: Coronavirus disease 2019
Cst-rs: Static compliance of the respiratory system
IMV: Invasive mechanical ventilation
PEEP: Positive end-expiratory pressure
